# Digoxin and exercise effects on skeletal muscle Na^+^,K^+^‐ATPase isoform gene expression in healthy humans

**DOI:** 10.1113/EP091962

**Published:** 2024-09-02

**Authors:** Michael J. McKenna, Xiaofei Gong, Aaron C. Petersen, Simon Sostaric, Craig A. Goodman, Andrew Garnham, Tai‐Juan Aw, Collene H. Steward, Kate T. Murphy, Kate A. Carey, Henry Krum, Rodney J. Snow, David Cameron‐Smith

**Affiliations:** ^1^ Institute for Health and Sport Victoria University Melbourne Australia; ^2^ Centre for Muscle Research, Department of Anatomy and Physiology University of Melbourne Melbourne Australia; ^3^ Department of Epidemiology and Preventive Medicine Monash University Alfred Hospital Melbourne Australia; ^4^ Institute for Breathing and Sleep Melbourne Australia; ^5^ Institute of Physical Activity and Nutrition, School of Exercise and Nutrition Sciences Deakin University Melbourne Australia; ^6^ Department of Nutrition Singapore Institute of Food and Biotechnology Innovation (SIFBI) Singapore Singapore

**Keywords:** digoxin, exercise, Na^+^,K^+^‐pump, mRNA, skeletal muscle

## Abstract

**Abstract:**

In muscle, digoxin inhibits Na^+^,K^+^‐ATPase (NKA) whereas acute exercise can increase NKA gene expression, consistent with training‐induced increased NKA content. We investigated whether oral digoxin increased NKA isoform mRNA expression (qPCR) in muscle at rest, during and post‐exercise in 10 healthy adults, who received digoxin (DIG, 0.25 mg per day) or placebo (CON) for 14 days, in a randomised, double‐blind and cross‐over design. Muscle was biopsied at rest, after cycling 20 min (10 min each at 33%, then 67% ${\dot{V}}_{O_{2}\mathrm{peak}}$), then to fatigue at 90% ${\dot{V}}_{O_{2}\mathrm{peak}}$ and 3 h post‐exercise. No differences were found between DIG and CON for NKA α_1–3_ or β_1–3_ isoform mRNA. Both α_1_ (354%, *P* = 0.001) and β_3_ mRNA (*P* = 0.008) were increased 3 h post‐exercise, with α_2_ and β_1–2_ mRNA unchanged, whilst α_3_ mRNA declined at fatigue (−43%, *P* = 0.045). In resting muscle, total β mRNA (∑(β_1_+β_2_+β_3_)) increased in DIG (60%, *P* = 0.025) and also when transcripts for each isoform were normalised to CON then either summed (*P* = 0.030) or pooled (*n* = 30, *P* = 0.034). In contrast, total α mRNA (∑(α_1_+α_2_+α_3_), *P* = 0.348), normalised then summed (*P* = 0.332), or pooled transcripts (*n* = 30, *P* = 0.717) did not differ with DIG. At rest, NKA α_1–2_ and β_1–2_ protein abundances were unchanged by DIG. Post‐exercise, α_1_ and β_1–2_ proteins were unchanged, but α_2_ declined at 3 h (19%, *P* = 0.020). In conclusion, digoxin did not modify gene expression of individual NKA isoforms at rest or with exercise, indicating NKA gene expression was maintained consistent with protein abundances. However, elevated resting muscle total β mRNA with digoxin suggests a possible underlying β gene‐stimulatory effect.

**Highlights:**

- **What is the central question of this study?**
  Na^+^,K^+^‐ATPase (NKA) in muscle is important for Na^+^/K^+^ homeostasis. We investigated whether the NKA‐inhibitor digoxin stimulates increased NKA gene expression in muscle and exacerbates NKA gene responses to exercise in healthy adults.
- **What is the main finding and its importance?**
  Digoxin did not modify exercise effects on muscle NKA α_1–3_ and β_1–3_ gene transcripts, which comprised increased post‐exercise α_1_ and β_3_ mRNA and reduced α_3_ mRNA during exercise. However, in resting muscle, digoxin increased NKA total β isoform mRNA expression. Despite inhibitory‐digoxin or acute exercise stressors, NKA gene regulation in muscle is consistent with the maintenance of NKA protein contents.

## 1 INTRODUCTION

In skeletal muscle, the Na^+^,K^+^‐ATPase (NKA) actively transports Na^+^ and K^+^ across sarcolemmal and transverse tubular membranes, thereby regulating transcellular Na^+^ and K^+^ gradients, membrane excitability and affecting muscle fatigue (Hostrup et al., 2021; Lindinger & Cairns, 2021; McKenna et al., 2024; Renaud et al., 2023). Given this essential role in muscle function, it is vitally important to understand NKA regulation, including by endogenous, stimulatory physiological factors such as exercise and also by exogenous, inhibitory pharmacological factors such as cardiotonic steroids (cardiac glycosides). NKA comprises a multi‐gene family, encoding a catalytic α subunit, regulatory β subunit and FXYD accessory protein, each with multiple isoforms, specific localisation and function and differing expression in muscle (Fedosova et al., 2021; Geering, 2006; McKenna et al., 2024; Yap et al., 2021). The *ATP1A1–3* and *ATP1B1–3* genes encode the NKA α_1–3_ and β_1–3_ isoform proteins and human muscle expresses gene transcripts of the α_1_–α_4_ and β_1_–β_3_ isoforms, with each readily and reliably detected except α_4_ (McKenna et al., 2024; Murphy et al., 2004; Murphy, Petersen, et al., 2006; Nordsborg et al., 2003, 2005).

Earlier studies reported exercise stimulated the expression of multiple genes in muscles (Hargreaves & Cameron‐Smith, 2002; Williams & Neufer, 1996). The regulation of NKA genes in muscle is complex, particularly with exercise (Christiansen, 2019; McKenna et al., 2024). Whilst the mechanisms responsible are incompletely understood, these potentially include acute increases in reactive oxygen species, intracellular [Na^+^], cytosolic [Ca^2+^] and reduced membrane potential (Christiansen, 2019). In rats, treadmill running increased α_1_ mRNA in the soleus muscle and β_2_ mRNA in extensor digitorum longus (EDL) and white gastrocnemius muscles (Tsakiridis et al., 1996), whereas electrical stimulation of EDL muscle increased each of α_1–3_ but not β_1–3_ mRNA at 3 h post‐contractions (Murphy, Macdonald, et al., 2006; Murphy et al., 2008). In human muscle, exercise‐induced changes in α_1–3_ gene transcripts, as well as their timing between 0 and 24 h post‐exercise, varied considerably, with increased α_1_, α_2_ or α_3_ mRNA respectively reported in seven, five and four of eight studies that utilised short, exhaustive or high‐intensity intermittent exercise (Aughey et al., 2007; Christiansen et al., 2018; Murphy et al., 2004; Nordsborg et al., 2003, 2005; Petersen et al., 2005) or prolonged cycling (Murphy, Petersen, et al., 2006; Murphy et al., 2008). Less consistent exercise effects on β_1–3_ mRNA were found, with respective increases in two, four and two of seven of these studies. A database accompanying a recent meta‐analysis of muscle transcriptomic responses to exercise in humans (Pillon et al., 2020) (https://metamex.serve.scilifelab.se/app/metamex) revealed significantly increased NKA α_1_ (31%) and β_3_ (68%) at 0–3 h after aerobic exercise and α_1_ (48%), β_1_ (14%) and β_3_ (27%) after resistance exercise. Collectively, these studies suggest a role for transcriptional regulation of multiple NKA genes in muscle after intense or prolonged contractions, with α_1_ mRNA consistently increased but variable effects on other genes. In red (oxidative) muscle from rats, a greater molar abundance of NKA β than α subunit proteins was found in purified plasma membranes (4:1) and intracellular membranes (5:2), with greater NKA activity evident in membrane fractions having a greater excess of β over α subunits (Lavoie et al., 1997). In human muscle, the mRNA expression of β_1_ was greater than of α_2_ (3.4:1) (Nordsborg et al., 2005). One possibility is therefore that α and β genes are differently affected by exercise, with upregulation in α mRNA more likely, consistent with the less abundant α than β subunit proteins. We investigated here whether exercise that comprised submaximal as well as exhaustive, near‐maximal intensity bouts increased muscle NKA gene transcripts.

Increased NKA gene responses to exercise may be a precursor to the robust increases in muscle NKA total content (total α protein, [^3^H]ouabain binding site content) found after training and with more variable reported increases in protein abundances of the individual NKA isoforms (Christiansen, 2019; McKenna et al., 2024; Wyckelsma et al., 2019). Several studies reported that acute exercise does not elevate the protein abundances of the NKA α_1–2_ or β_1–2_ isoforms (Aughey et al., 2007; Green et al., 2011; Murphy et al., 2004; Murphy, Petersen, et al., 2006), whereas an extreme, 16 h interval exercise session increased α_2_ protein (Green et al., 2007). Whilst changes in NKA isoform protein abundances were not anticipated with acute exercise, these were also determined here.

Cardiotonic steroids such as ouabain and digoxin specifically bind to and inhibit NKA activity in mammalian muscle, including in humans (Bonting et al., 1961; Clausen & Hansen, 1974; Nielsen & Clausen, 1997; Nørgaard et al., 1984) and may therefore also affect NKA gene expression. In rat kidney outer medullary tubules, 10^−4^ M ouabain doubled α_1_ and β_1_ mRNA after 1–2 h, which remained elevated for 9 h and with gene transcription rates increased for the first hour (Rayson, 1993). In rats, 1 mM ouabain did not modify α_1–3_ or β_1_ mRNA in EDL muscle, but reduced β_2_ and β_3_ mRNA by 76% and 92%, respectively (Murphy, Macdonald, et al., 2006). In human muscle, after prolonged exhaustive exercise, a reduction in the maximal in vitro K^+^‐stimulated 3‐*O*‐methylfluorescein phosphatase (3‐*O*‐MFPase) activity used as a marker of NKA activity (McKenna et al., 2008), was correlated with increased post‐exercise α_1_ and α_2_ mRNA, suggesting a link in human muscle between reduced NKA activity and increased NKA gene expression (Petersen et al., 2005). Whilst this decline in 3‐*O*‐MFPase activity after exercise in human muscle is consistent (McKenna et al., 2008), variable findings exist on whether directly measured NKA activity is also depressed (McKenna et al., 2024). In humans, digoxin is used to treat patients with severe heart failure and atrial fibrillation (Angraal et al., 2019; Bavendiek et al., 2017). In these patients, digoxin was found to occupy a small fraction (∼9–13%) of NKA in their muscle, which therefore lowered the measured [^3^H]ouabain binding site content (Schmidt & Kjeldsen, 1991; Schmidt, Holm‐Nielsen, et al., 1993; Schmidt et al., 1995). Whether inhibition by digoxin also affects NKA gene expression in human muscle at rest and/or with exercise is unknown, with differing effects of digoxin on gene expression in different tissues in other species. Such an effect might be mediated by greater increases induced by digoxin in intracellular [Na^+^] with exercise and stimulation of NKA synthesis. Cardiotonic steroid effects are of further potential importance since their inhibition of NKA α_1_ also modulates intracellular signalling in other tissues (Aperia et al., 2016; Cui & Xie, 2017) whilst in gene‐targeted mice lacking one copy of α_1_ (α_1_+/−) where the muscle α_1_ abundance was reduced by 30–34%, soleus muscle growth was impaired by 9% (Kutz et al., 2018). We therefore also investigated the effects of digoxin on NKA isoform gene expression in human muscle.

The purpose of this study was to quantify the effects of digoxin taken orally for 14 days and of acute fatiguing exercise on NKA isoform mRNA expression and protein abundances in skeletal muscle in healthy humans. We measured NKA α_1–3_ and β_1–3_ isoform mRNA expression by RT‐PCR and NKA α_1–2_ and β_1–2_ protein abundances by western blotting. We tested the hypotheses in healthy humans that acute exercise would increase NKA α and β mRNA expression and that oral digoxin intake would increase NKA α and β mRNA expression in muscle at rest and after exercise.

## 2 METHODS

### 2.1 Ethics approval

The study was approved by the Victoria University Human Research Ethics Committee (VU HREC 01/100; HREC 02/57; HREC 02/105) and the Alfred Hospital Ethics Committee (Project no. 72/03) and conforms to the principles outlined in the *Declaration of Helsinki*, except for registration in a database. All participants in the study gave written informed consent.

### 2.2 Study design and participants

This paper forms part of a larger study investigating digoxin effects on human muscle NKA content, activity, K^+^ homeostasis and muscle function, with all methods and findings fully detailed elsewhere (Sostaric et al., 2022). Here, we report digoxin and exercise effects on muscle NKA isoform gene expression and protein abundance in 10 recreationally active young adults (one female, nine males; age, 26.1 (5.9) years; height, 178.4 (9.1) cm; body mass, 75.7 (11.3) kg; ${\dot{V}}_{O_{2}\mathrm{peak}}$, 3.67 (0.42) l min^−1^; mean (SD)). Trials were conducted in a randomised, double‐blind, crossover, counterbalanced design with participants randomly allocated to either a digoxin (DIG) or placebo (CON) treatment group and given either digoxin at 0.25 mg per day (Lanoxin, Glaxo Smith Kline, London, UK) or a placebo (sugar tablet) for 14 days and underwent an exercise test and muscle biopsies. After a 4‐week washout period, the groups switched to the alternative treatment for a further 14 days and then repeated the exercise test and biopsies. Thus, for those participants taking DIG first, the effective digoxin clearance time was 6 weeks, that is, the standard 4‐week washout plus the 2 weeks of placebo treatment. Similarly, for the placebo group, the time between biopsies was 6 weeks. This washout period should be more than sufficient, as the digoxin clearance half‐time from serum after digoxin injection was 45 h (range 32–131 h) (Kramer et al., 1974) and from skeletal muscle after oral digoxin was ∼2.2 days (Jogestrand & Sundqvist, 1981). The serum [digoxin] at rest was elevated at 14 days in DIG to 0.8 (0.2) nM, whilst in CON, [digoxin] was below or at the detection limit in all participants (Sostaric et al., 2022).

### 2.3 Cycle exercise test and muscle biopsy sampling

Participants consumed pre‐packaged iso‐energetic meals and beverages during 72 h before each experimental trial and refrained from vigorous activity and ingestion of caffeine and alcohol in the 24 h before each visit (Sostaric et al., 2022). Participants were also verbally instructed to maintain their habitual physical activity patterns throughout the study. These minimised the likelihood of any changes in physical activity or dietary influences on muscle NKA content or gene expression. Participants cycled for 10 min at 33% ${\dot{V}}_{O_{2}\mathrm{peak}}$ followed by 2 min rest, 10 min at 67% ${\dot{V}}_{O_{2}\mathrm{peak}}$, followed by 2 min rest and then continued cycling until fatigue at 90% ${\dot{V}}_{O_{2}\mathrm{peak}}$ on day 14 of both trials. A muscle biopsy was taken at rest, immediately after exercise at 67% ${\dot{V}}_{O_{2}\mathrm{peak}}$ and at fatigue, and at 3 h post‐exercise, with timing of the latter to facilitate detection of any changes in NKA gene transcript expression (Murphy et al., 2004; Nordsborg et al., 2003).

A local anaesthetic (1% lidocaine) was injected into the skin and subcutaneous tissue over the vastus lateralis muscle, four small incisions (2 per leg) were made through the skin and fascia, and a muscle sample of approximately 100–120 mg was then excised using a biopsy needle. Samples were immediately frozen in liquid N_2_ until assayed later for NKA isoform mRNA expression and protein abundance and also for [^3^H]ouabain binding site content, digoxin occupancy and maximal in vitro K^+^‐stimulated 3‐*O*‐MFPase activity, as reported elsewhere (Sostaric et al., 2022).

### 2.4 Real‐time RT‐PCR measurement of mRNA

Total RNA was extracted from 5–10 mg muscle and transcribed into cDNA using methods previously employed in our laboratory (Murphy et al., 2003). Gene expression of the Na^+^,K^+^‐ATPase α_1,_ α_2_, α_3_, β_1_, β_2_ and β_3_ isoforms was quantified using quantitative real‐time RT‐PCR (qPCR), using previously described methods (Murphy et al., 2004; Murphy, Petersen, et al., 2006). Total RNA was extracted from 5–10 mg muscle using the FastRNA reagents (BIO 101, Vista, CA, USA) (Cameron‐Smith et al., 2003; Murphy et al., 2001, 2003). The resulting RNA pellet was dissolved in EDTA‐treated water and stored at −80°C. Total RNA concentration was determined spectrophotometrically at 260 nm. For each sample, 1 µg of RNA was transcribed into cDNA using the Promega AMV Reverse Transcription Kit (kit A3500; Promega, Madison, WI, USA), and the resulting cDNA was stored at −20°C for subsequent analysis. Real Time‐PCR (GeneAmp 7500 sequence detection system; Thermo Fisher Scientific, Waltham, MA, USA) was run for 1 cycle (50°C for 2 min and 95°C for 10 min) and 50 cycles (95°C for 15 s and 60°C for 60 s). Fluorescence resulted from incorporation of SYBR Green (SYBR Green Master Mix, Thermo Fisher Scientific) to double stranded DNA and this fluorescence was measured after each repetitive cycle. Duplicate wells were run for each sample. Measurements included a no‐template control, as well as a human muscle sample endogenous control, cyclophilin (CYC). Primer sequences were designed from published sequences, where possible spanning exon boundaries to minimise contaminant DNA amplification (Table 1). The relative expression of the genes was normalised for input cDNA using the housekeeping gene *cyclophilin* and were therefore expressed as $2^{-\Delta CT}$. Neither exercise (*P* = 0.82) nor digoxin (*P* = 0.64) had any significant effect on the mRNA expression of *cyclophilin*, when expressed in the linear ($2^{-\Delta CT}$) form (data not shown). Intra‐assay variability of $2^{-\Delta CT}$ values for respective isoforms was (coefficient of variation; CV, %): α_1_ 13.8; α_2_ 10.6; α_3_ 12.5; β_1_ 8.9; β_2_ 12.7; β_3_ 14.0; human CYC 8.6 – all within values previously reported (Murphy et al., 2003). All $2^{-\Delta CT}$ data are reported after being normalised relative to the mean of the placebo trial resting samples, thus also demonstrating variability of resting mRNA samples.

**TABLE 1:** Human Na^+^,K^+^‐ATPase gene α_1_‐α_3_ and β_1_‐β_3_ and CYC primer sequences used for mRNA analyses.

Gene	GenBank accession	Identity	Sense primer (5′−3′)	Antisense primer (5′−3′)	Exon boundaries
α_1_	NM_000701	ATPA1	TGTCCAGAATTGCAGGTCTTTG	TGCCCGCTTAAGAATAGGTAGGT	4
α_2_	NM_000702	ATPA2	GAATGAGAGGCTCATCAGCAT	CAAAGTAGGTGAAGAAGCCACC	12‐13
α_3_	NM_152296	ATPA3	GGTGGCTATGACAGAGCACAA	TGCACACAGTGTGTGTTGTATTT	1‐3
β_1_	NM_001677	ATPB1	ACCAATCTTACCATGGACACTG	CGGTCTTTCTCACTGTACCCAAT	3‐6
β_2_	NM_001679	ATPB2	CCAGCATGTTCAGAAGCTCAAC	GCGGCAGACATCATTCTTTTG	4
β_3_	BC011835	ATPB3	AGTCTGTCCTGATGGAGCACTT	GCATGCTTGAAGTAATGAAATA	4
CYC	XM_004890	PPIA	CCCACCGTGTTCTTCGACAT	CCAGTGCTCAGAGCACGAAA	

*Note*: Primer sequences were designed using Applied Biosystems Primer Express software (Thermo Fisher Scientific) from gene sequences obtained from GeneBank. Primer specificity was determined using a BLAST search. Abbreviation: CYC, cyclophilin.

#### 2.4.1 Calculations for resting muscle data

Since qPCR is a relative measure, the total α mRNA and β mRNA expression in resting muscle in DIG and CON were calculated as the sum of the relative amounts ($2^{-\Delta CT}$) values of α_1_, α_2_ and α_3_ (∑(α_1_+α_2_+α_3_) mRNA, *n* = 10) and total β mRNA as the sum of the relative amounts ($2^{-\Delta CT}$) of β_1_, β_2_ and β_3_ (∑(β_1_+β_2_+β_3_) mRNA, *n* = 10). To reduce possible effects of intra‐ and inter‐individual variations for these comparisons, the relative amounts ($2^{-\Delta CT}$) of each isoform in resting muscle were also normalised for digoxin relative to control and the sum of these normalised mRNA was then calculated for each individual for α (∑ (normalised α_1_ + normalised α_2_ + normalised α_3_ mRNA), *n* = 10) and for β mRNA (∑ (normalised β_1_ + normalised β_2_ + normalised β_3_ mRNA), *n* = 10) and compared against control (normalised and summed). To further assess whether an overall change in α or β mRNA could be detected, each normalised data point for the three α or β isoforms was also considered separately and the pooled data (*n* = 30, i.e., each of the rest, 67% ${\dot{V}}_{O_{2}\mathrm{peak}}$, fatigue and +3 h post‐exercise samples) contrasted against control for all normalised α mRNA and normalised β mRNA (normalised and pooled). Similar comparisons of summed mRNA responses for exercise effects were not conducted for α or β isoforms due to the considerable variations reported in individual isoform responses to exercise (Aughey et al., 2007; Christiansen et al., 2018; Murphy et al., 2008, 2004; Murphy, Petersen, et al., 2006; Nordsborg et al., 2003, 2005; Petersen et al., 2005; Pillon et al., 2020).

### 2.5 Western blotting

Approximately 10–20 mg of frozen muscle sample was used for NKA immunoblot analyses. Muscle proteins were lysed in ice‐cold buffer containing 20 mM Tris pH 7.8, 137 mM NaCl, 2.7 mM KCl, 1 mM MgCl_2_, 5 mM Na_4_O_7_P_2_, 10 mM NaF, 1% Triton X‐100, 10% glycerol, 1% Protease Inhibitor Cocktail (P8340 Sigma‐Aldrich, Castle Hill, NSW, Australia). Samples were homogenised (1:60 dilution) using a tissue Lyser II (Qiagen, Hilden, Germany) followed by gentle rocking for 60 min at 4°C. Protein concentration of the homogenate was determined using a commercially available kit (DC Protein Assay, Bio‐Rad Laboratories, Hercules, CA, USA). Repeated steps of centrifugation of muscle and membrane separation have resulted in very low recovery of NKA, yielding a final sample that may be unrepresentative of the whole muscle NKA (Hansen & Clausen, 1988). Therefore, muscle NKA isoform analyses did not include any membrane isolation steps, to maximise recovery of NKA enzymes (Murphy et al., 2004). Aliquots of the muscle homogenate were diluted to the same protein concentration using lysis buffer. Aliquots were then deglycosylated by incubating the homogenate for 1 h at 37°C with 0.5% (v/v) Nonidet P40 and 3 U *N*‐Glycosidase F (Boehringer Mannheim, North Ryde, Australia) per 0.5 mg protein. The muscle homogenate was mixed with Laemmli sample buffer and proteins were separated on 26 well Criterion Stain Free precast gels (8%–16% Criterion TGX, Bio‐Rad Laboratories) for 30 min at 80 V followed by 60–90 min at 120 V.

For the analysis of protein abundance of the NKA isoforms, 15 µg of total protein per sample was loaded in each lane for α_1_ and α_2_, and 6 µg for β_1_ and β_2_. The α_3_ and β_3_ isoforms could not be detected despite several attempts under varying conditions. To ensure that blot density was within the linear range of detection (Murphy & Lamb, 2013), a 4‐ to 5‐point (1.5–12 µg) calibration curve of whole‐muscle crude homogenate was loaded onto every gel. The homogenate was prepared from an equal amount of 5 µL from each sample. Following electrophoresis, proteins for NKA α isoform analyses were transferred to polyvinylidene fluoride membranes (TurboTransfer pack, Bio‐Rad Laboratories) for 10 min at 25 V using the semi‐dry Trans‐Blot Turbo Transfer System (Bio‐Rad Laboratories). Proteins for β isoform analyses were transferred to low‐fluorescence polyvinylidene fluoride membranes (TurboTransfer pack, Bio‐Rad Laboratories) for 10 min at 25 V using the semi‐dry Trans‐Blot Turbo Transfer System (Bio‐Rad Laboratories). Membranes were blocked in TBST buffer (10 mM Tris, 100 mM NaCl, 0.02% Tween‐20) containing 5% non‐fat milk, for 1 h at room temperature. After being washed (4 × 4 min in TBST), membranes were incubated with the appropriate primary antibody overnight at 4°C. The following antibodies were used for NKA isoform α_1_ (monoclonal α6F (1:750), developed by D. Fambrough, obtained from the Developmental Studies Hybridoma Bank, maintained by the University of Iowa, USA), α_2_ (polyclonal no. 07‐674 (1:500), Merck Millipore, Burlington, MA, USA), β_1_ (monoclonal no. 05‐382 (1:500), Thermo Fisher Scientific), β_2_ (polyclonal no. 22338‐1‐AP (1:500), Proteintech, Rosemont, IL, USA). All antibodies have previously been validated for human skeletal muscle (Christiansen et al., 2018). Primary antibodies were diluted in TBST buffer containing 0.1% NaN_3_ and 1% bovine serum albumin. Following incubation with the primary antibodies, membranes were washed in TBST buffer (4 × 4 min) and incubated for 1 h at room temperature with anti‐mouse (PerkinElmer (Waltham, MA, USA) no. NEF822001EA for α_1_; Cell Signaling Technology (Danvers, MA, USA) no. 7076 for β_1_) or anti‐rabbit (PerkinElmer no. NEF812001EA for α_2_; Cell Signaling Technology no. 7074 for β_2_) horseradish peroxidase‐conjugated secondary antibodies diluted 1:5000 for α_1_ and 1:20000 for α_2_, β_1_ and β_2_ in TBST with 5% non‐fat milk. After washing the membranes in TBST (4 × 4 min), membranes were incubated for 5 min with SuperSignal West Femto Maximum Sensitivity Substrate (Thermo Fisher Scientific), then stain free images were taken using a ChemiDoc Imaging system (Bio‐Rad Laboratories). The densities of samples were expressed relative to the total protein on the membrane and then normalised to the calibration curve (Murphy & Lamb, 2013). All protein data were reported after being normalised relative to the mean of the placebo trial resting samples.

### 2.6 Statistical analyses

All data are presented as means (SD). Data were first tested for normality using the Shapiro–Wilk test and if criteria were not met, data were log‐transformed. Data sets were analysed using a linear mixed model, with time (rest, 67% ${\dot{V}}_{O_{2}\mathrm{peak}}$, fatigue, 3 h recovery) and treatment (DIG, CON) as fixed effects, and restricted maximum likelihood as the estimation method for missing values. Post‐hoc analyses used the least significant difference test. Significant treatment effects are reported, with *P*‐values also reported for non‐significant treatment effects. Where significant time effects were found, differences between times are indicated, but non‐significant differences between times are not detailed. To avoid repetition, treatment‐by‐time interactions are only stated where significant. Paired raw data in resting muscle for the total α or β subunit mRNA expression were analysed using Student's *t*‐test for paired data. Total α or β gene transcripts normalised against control were log‐transformed and then analysed using a one sample *t*‐test to determine difference from the set value equal to the placebo value. Sample size was *n* = 10 for all isoform gene expression, whilst for western blots, was *n* = 5 for α isoforms and *n* = 7 for β isoform measures, due to inadequate tissue for some samples. Statistical significance was accepted at *P* ≤ 0.05 and observed statistical power was as reported by SPSS for treatment and time effects (Supporting information, Table S1). Statistical analyses were performed using IBM SPSS Statistics 27 (IBM Corp., Armonk, NY, USA); since this package does not report exact *P*‐values lower than *P *< 0.001, these data are reported here as *P* = 0.001.

## 3 RESULTS

### 3.1 NKA α mRNA expression

#### 3.1.1 Digoxin

**FIGURE 1:**
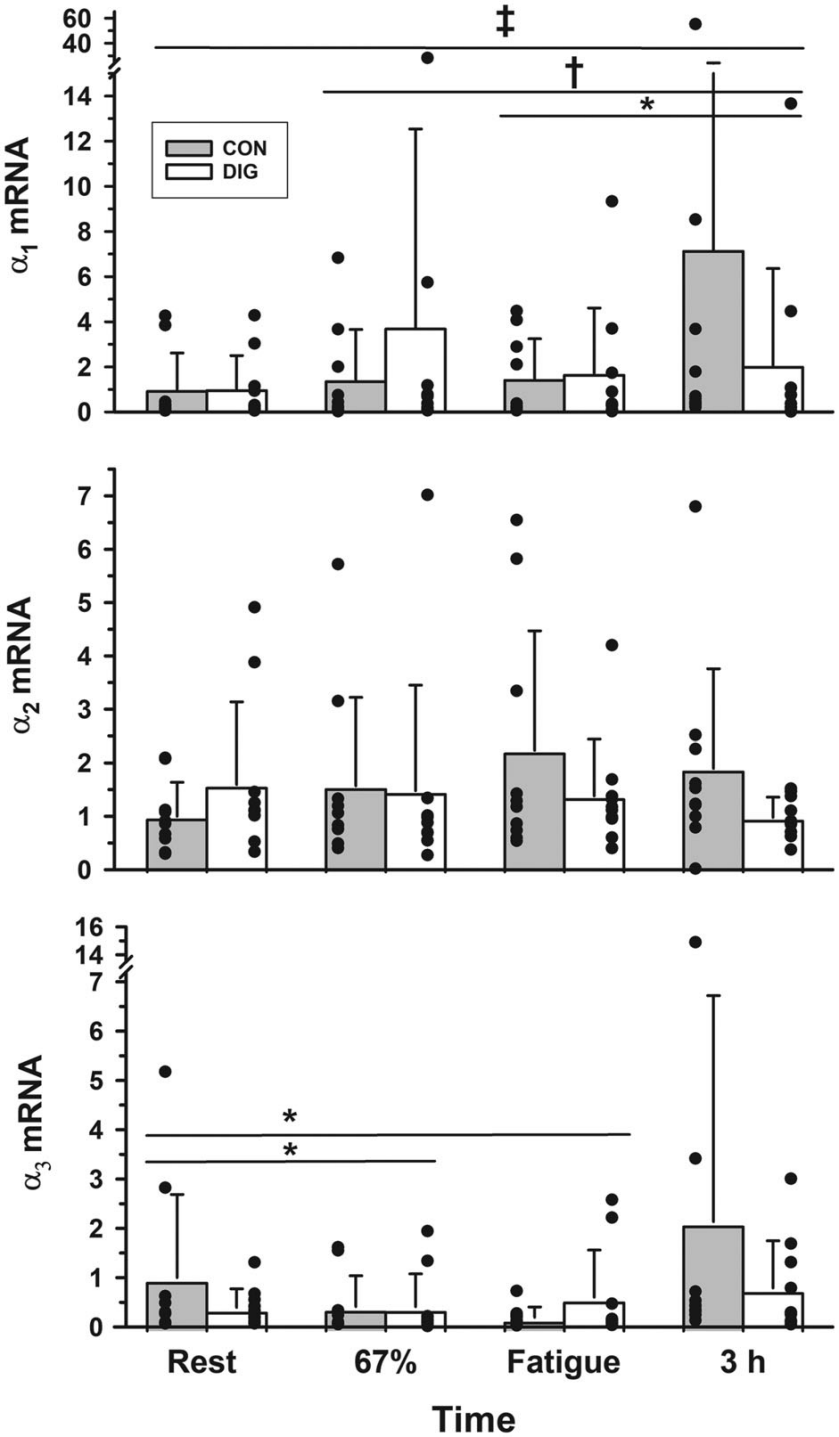
The effects of digoxin and intense cycling exercise on skeletal muscle Na^+^,K^+^‐ATPase α isoform mRNA expression. Data expressed as mean (SD), *n* = 10 for all isoform mRNA. Individual data are shown by filled circles for each measurement. Samples were collected at rest, after 20 min cycling exercise comprising 10 min at 33% ${\dot{V}}_{O_{2}\mathrm{peak}}$ then 10 min at 67% ${\dot{V}}_{O_{2}\mathrm{peak}}$, immediately following cycling at 90% ${\dot{V}}_{O_{2}\mathrm{peak}}$ continued to fatigue, and at 3 h post‐exercise (+3 h). Isoform gene expression was normalised to the housekeeping gene *cyclophilin* and expressed as $2^{-\Delta CT}$. All $2^{-\Delta CT}$ data are reported normalised relative to the mean of the placebo trial resting samples. Time effects are indicated by a horizontal line between times that differed from rest or from other times: *P < 0.05, †*P *< 0.01, ‡*P *< 0.001. There were no significant treatment (DIG) effects for α_1_ (*P* = 0.648), α_2_ (*P* = 0.710) and α_3_ (*P* = 0.707). Time × treatment interactions were not significant.

None of the α_1_ mRNA (*P* = 0.648), α_2_ mRNA (*P* = 0.710) or α_3_ mRNA (*P* = 0.707) differed between DIG and CON (Figure 1).

#### 3.1.2 Exercise

The α_1_ mRNA increased above rest at 3 h post‐exercise (354%, time effect, *P* = 0.001), where it was also greater than at 67% ${\dot{V}}_{O_{2}\mathrm{peak}}$ (79%, *P* = 0.008) and at fatigue (189%, *P* = 0.022; Figure 1). In contrast, there was no effect of exercise on α_2_ mRNA (*P* = 0.631), whilst the α_3_ mRNA fell below rest at 67% ${\dot{V}}_{O_{2}\mathrm{peak}}$ (−41%, *P* = 0.021) and at fatigue (−43%, *P* = 0.045), but had returned to rest at 3 h recovery (*P* = 0.100; Figure 1).

### 3.2 NKA β mRNA expression

#### 3.2.1 Digoxin

**FIGURE 2:**
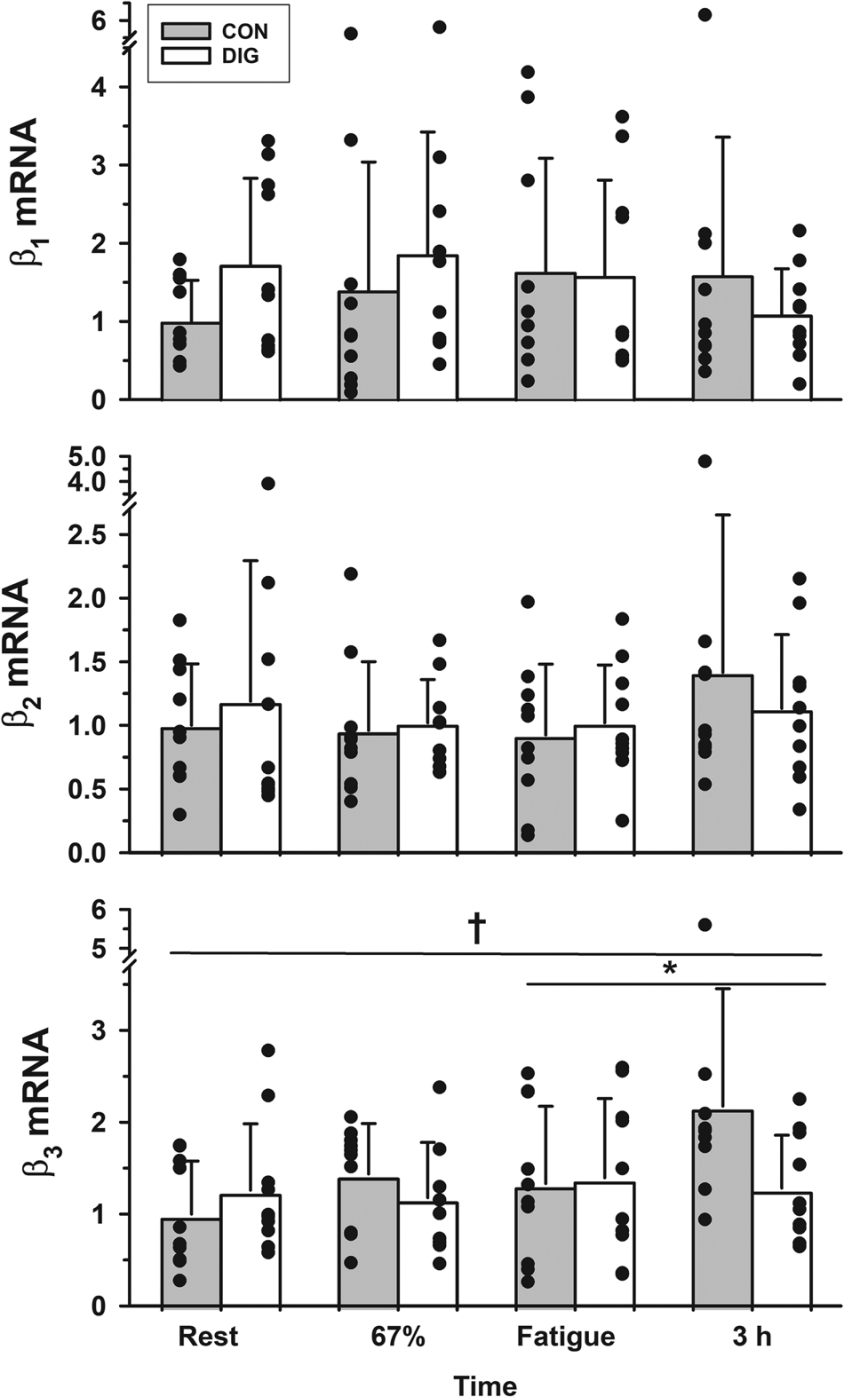
The effects of digoxin and intense cycling exercise on skeletal muscle Na^+^,K^+^‐ATPase β isoform mRNA expression. Data expressed as means (SD), *n* = 10 for all isoform mRNA. Individual data are shown by filled circles for each measurement. Samples were collected at rest, after 20 min cycling exercise comprising 10 min at 33% ${\dot{V}}_{O_{2}\mathrm{peak}}$ then 10 min at 67% ${\dot{V}}_{O_{2}\mathrm{peak}}$, immediately following cycling at 90% ${\dot{V}}_{O_{2}\mathrm{peak}}$ continued to fatigue, and at 3 h post‐exercise (+3 h). Isoform gene expression was normalised to the housekeeping gene *cyclophilin* and expressed as $2^{-\Delta CT}$. All $2^{-\Delta CT}$ data are reported normalised relative to the mean of the placebo trial resting samples. Time effects are indicated by a horizontal line between times that differed from rest or from other times: *P < 0.05, †*P *< 0.01. There were no significant treatment (DIG) effects for β_1_ (*P* = 0.115), β_2_ (*P* = 1.000) or β_3_ mRNA (*P* = 0.401). Time × treatment interactions were not significant.

None of the β_1_ mRNA (*P* = 0.115), β_2_ mRNA (*P* = 1.000) or β_3_ mRNA (*P* = 0.401) differed between DIG and CON (Figure 2).

#### 3.2.2 Exercise

There were no effects of exercise or recovery on β_1_ mRNA (*P* = 0.913) or β_2_ mRNA (*P* = 0.109, Figure 2), whilst β_3_ mRNA trended toward significance (*P* = 0.051, time effect) and was elevated at 3 h post‐exercise above both rest (53%, *P* = 0.008) and fatigue (27%, *P* = 0.042, Figure 2).

### 3.3 NKA total mRNA expression in rest muscle

#### 3.3.1 Total α mRNA in rest muscle

**FIGURE 3:**
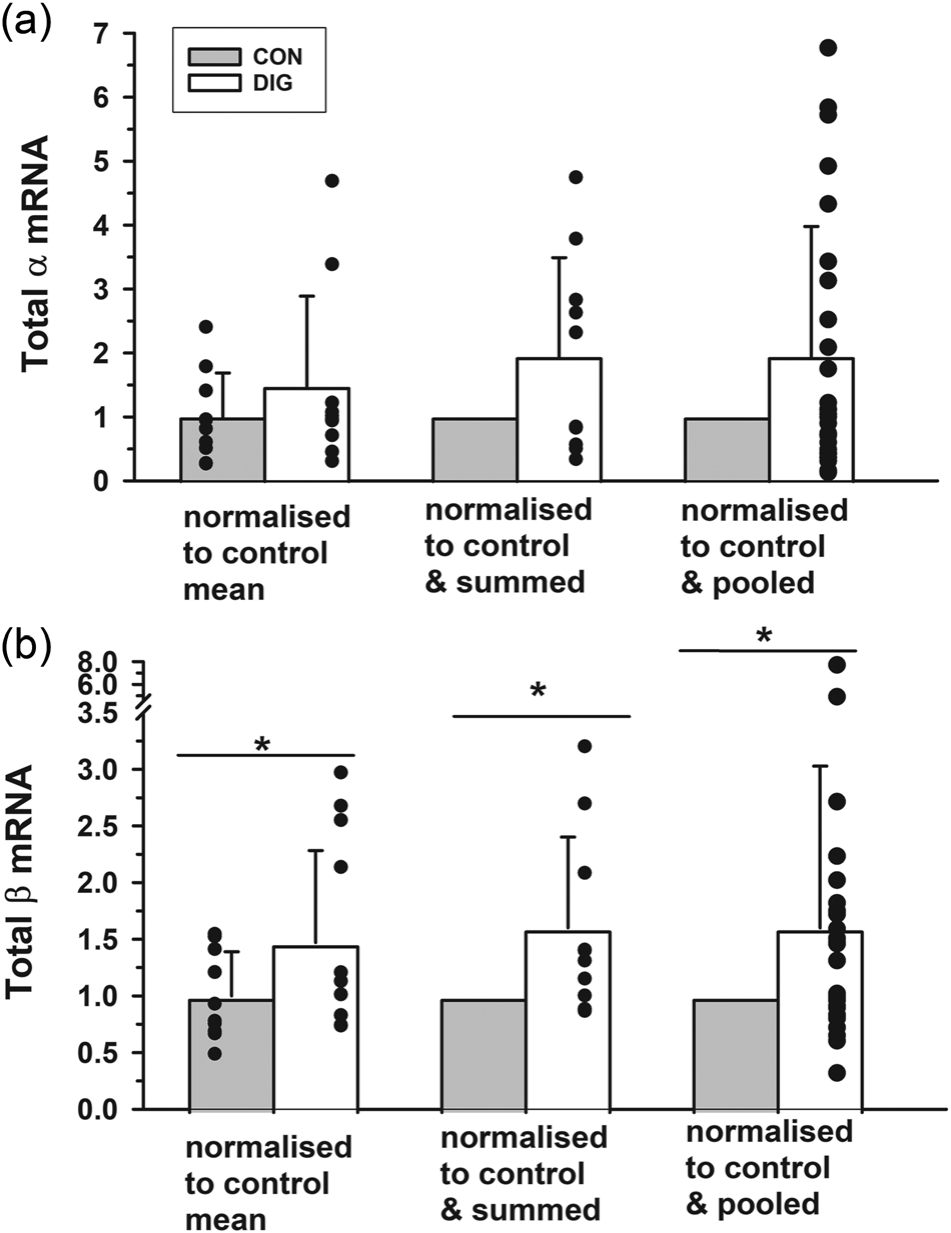
The effects of digoxin on Na^+^,K^+^‐ATPase (a) total α and (b) total β isoform mRNA expression in resting skeletal muscle. Data expressed as means (SD). Individual data are shown by filled circles. Left panels: the relative expression of each isoform was normalised to the housekeeping gene *cyclophilin* and expressed as $2^{-\Delta CT}$, and data were then summed (*n* = 10, i.e., α_1_+α_2_+α_3_ (a) and β_1_+β_2_+β_3_ (b)) to give relative total subunit expression. The summed data are reported normalised relative to the mean of the placebo trial resting samples. Differences between digoxin and control were analysed by Student's paired *t*‐test. Middle panels: the $2^{-\Delta CT}$ data for each isoform mRNA were normalised to control (i.e., each individual control equalled 1.0) and data were then summed (*n* = 10, i.e., α_1_+α_2_+α_3_ (a) and β_1_+β_2_+β_3_ (b)), with digoxin analysed using a one sample *t*‐test. Individual data are shown only for the digoxin data. Right panels: the mRNA $2^{-\Delta CT}$ data for each isoform were normalised to control (1.0) and then all data were pooled (*n* = 30, i.e., α_1_, α_2_, α_3_ each *n* = 10 (a) and β_1_, β_2_, β_3_ each *n* = 10 (b)), with digoxin analysed using a one sample *t*‐test. Individual data are shown only for the digoxin data. Differences between digoxin and control are indicated by a horizontal line: *P < 0.05.

Additional calculations to determine the possible effects of digoxin on total NKA mRNA expression were performed in resting muscle only. The summed raw α mRNA expression data (∑(α_1_+α_2_+α_3_) mRNA, *n* = 10) did not differ between DIG and CON (*P* = 0.348, Figure 3). No difference was found for total α mRNA, when normalised relative to CON for each isoform and then either summed (*n* = 10, *P* = 0.332, Figure 3) or pooled (*n* = 30, *P* = 0.717, Figure 3).

#### 3.3.2 Total β mRNA in rest muscle

In resting muscle only, the summed raw β mRNA expression (∑(β_1_+β_2_+β_3_), *n* = 10) was 60% greater in DIG than in CON (*P* = 0.025, Figure 3). This increase in DIG was also apparent when β mRNA expression data for each isoform were normalised relative to CON and then either summed (*n* = 10, *P* = 0.030, Figure 3) or pooled (*n* = 30, *P* = 0.034, Figure 3).

### 3.4 NKA α and β protein abundances

**FIGURE 4:**
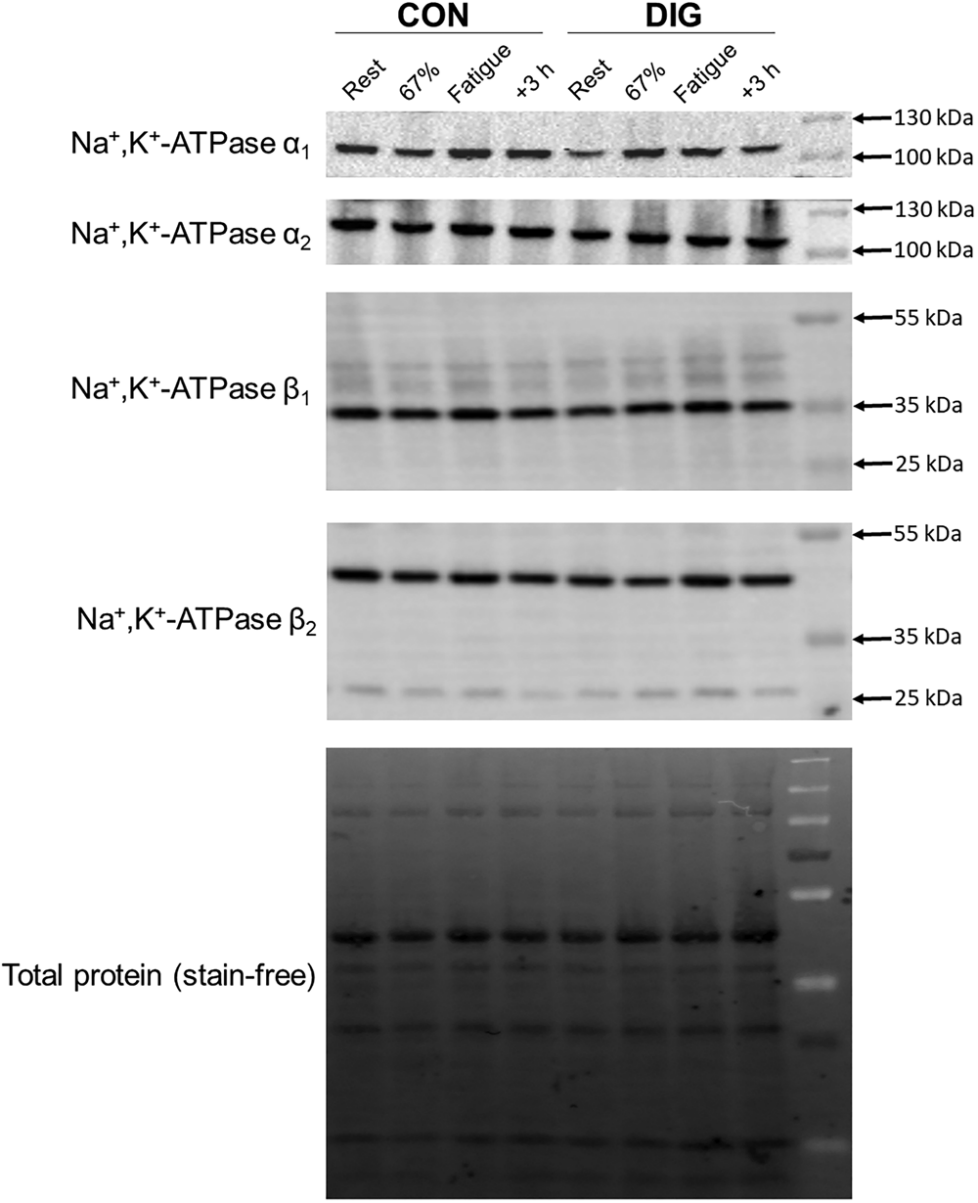
Representative western blots. Representative western blot images for NKA α and β isoform protein determinations in crude muscle homogenates after 14 days’ digoxin (DIG) or placebo (CON). Biopsies were taken at rest (Rest), after cycling for 20 min comprising 10 min at 33% ${\dot{V}}_{O_{2}\mathrm{peak}}$ then 10 min at 67% ${\dot{V}}_{O_{2}\mathrm{peak}}$ (67%), immediately following cycling at 90% ${\dot{V}}_{O_{2}\mathrm{peak}}$ continued to fatigue (Fatigue), and at 3 h post‐exercise (+3 h).

Representative blots for α and β protein measures in crude muscle homogenates are shown in Figure 4.

#### 3.4.1 Digoxin

**FIGURE 5:**
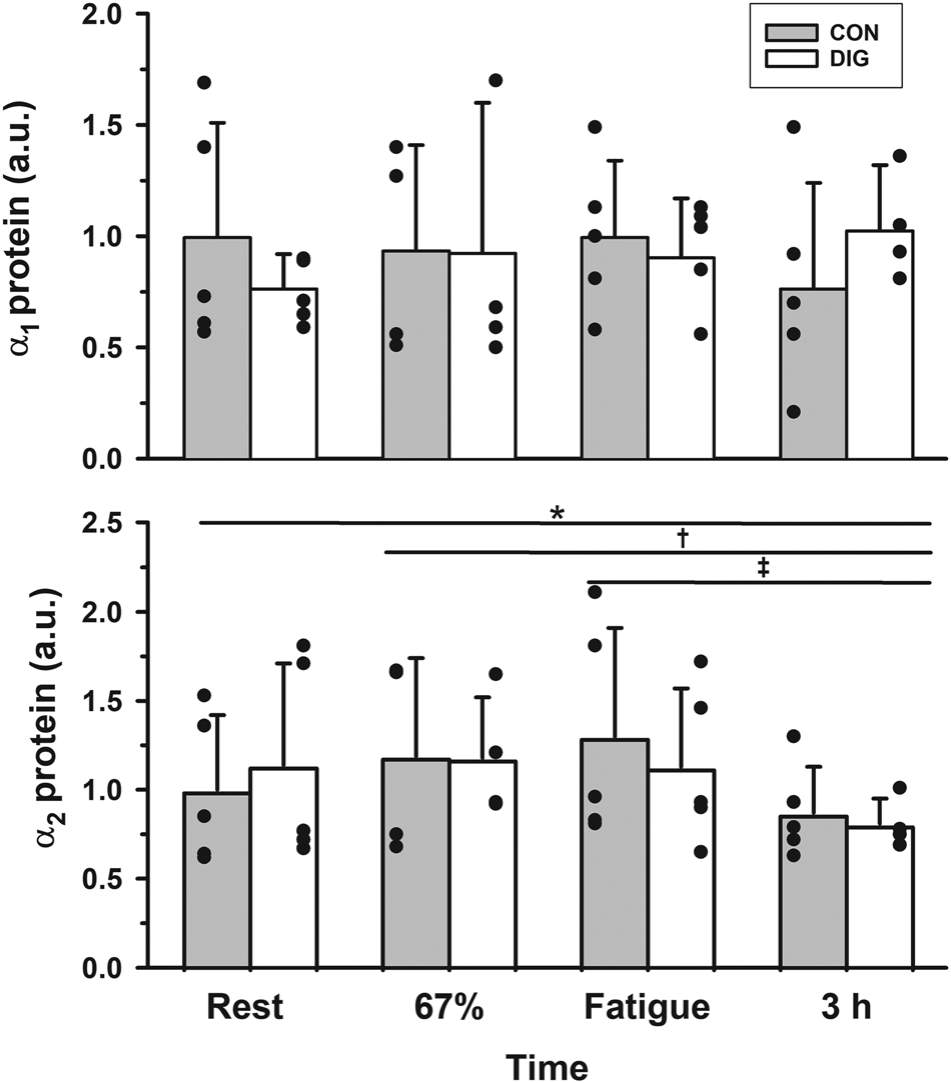
The effects of digoxin and intense cycling exercise on skeletal muscle Na^+^,K^+^‐ATPase α isoform protein abundances. Data expressed as means (SD) in arbitrary units (a.u.). Individual data are shown by filled circles for each measurement. Samples were collected at rest, after 20 min cycling exercise comprising 10 min at 33% ${\dot{V}}_{O_{2}\mathrm{peak}}$ then 10 min at 67% ${\dot{V}}_{O_{2}\mathrm{peak}}$, immediately following cycling at 90% ${\dot{V}}_{O_{2}\mathrm{peak}}$ continued to fatigue, and at 3 h post‐exercise (+3 h). Protein abundances were determined by western blotting for α_1_ and α_2_ (*n* = 5). All protein data are reported normalised relative to the mean of the placebo trial resting samples. Time effects are indicated by a horizontal line between times which differed from rest or from other times, *P = 0.02; †*P* = 0.01; ‡*P* = 0.001. There were no significant time effects for α_1_ or treatment (DIG) effects for α_1_ (*P* = 0.579) or α_2_ (*P* = 0.476). Time × treatment interactions were not significant for isoform protein abundances.

**FIGURE 6:**
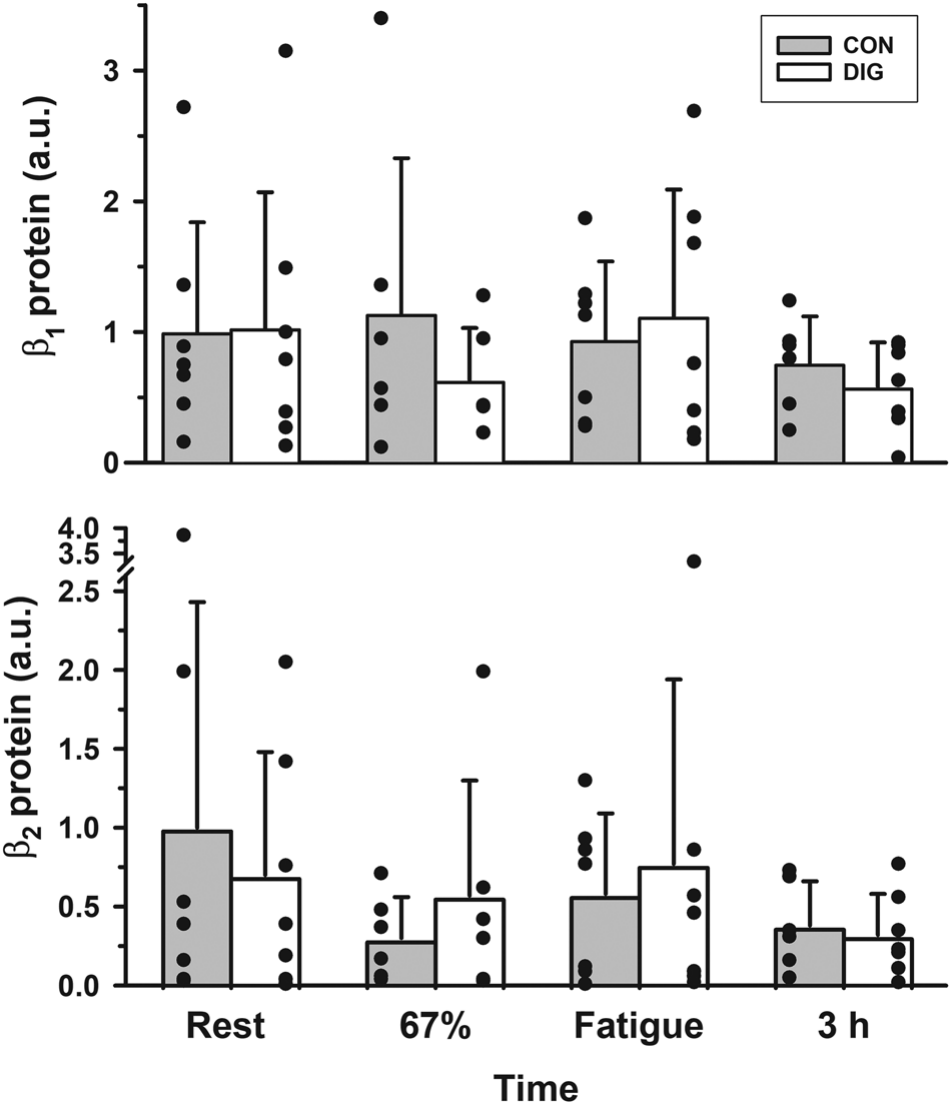
The effects of digoxin and intense cycling exercise on skeletal muscle Na^+^,K^+^‐ATPase β isoform protein abundances. Data expressed as means (SD) in arbitrary units (a.u.). Individual data are shown by filled circles for each measurement. Samples were collected at rest, after 20 min cycling exercise comprising 10 min at 33% ${\dot{V}}_{O_{2}\mathrm{peak}}$ then 10 min at 67% ${\dot{V}}_{O_{2}\mathrm{peak}}$, immediately following cycling at 90% ${\dot{V}}_{O_{2}\mathrm{peak}}$ continued to fatigue, and at 3 h post‐exercise (+3 h). Protein abundances were determined by western blotting for β_1_ and β_2_ (*n* = 7). All protein data are reported normalised relative to the mean of the placebo trial resting samples. There were no significant time or treatment (DIG) effects for β_1_ (*P* = 0.402) or β_2_ (*P* = 0.733). Time × treatment interactions were not significant for isoform protein abundances.

There were no effects of DIG on α_1_ (*P* = 0.579), α_2_ (*P* = 0.476) (Figure 5) or on β_1_ (*P* = 0.402) or β_2_ (*P* = 0.733) protein abundances (Figure 6).

#### 3.4.2 Exercise

There was no effect of exercise on the NKA α_1_ (*P* = 0.843), β_1_ (*P* = 0.565) or β_2_ (*P* = 0.989) protein abundances (Figures 5 and 6). The α_2_ protein abundance was unchanged during exercise, but at 3 h post‐exercise had declined below rest (−19%, *P* = 0.020), 67% ${\dot{V}}_{O_{2}\mathrm{peak}}$ (−33%, *P* = 0.010) and fatigue (−33%, *P* = 0.001, Figure 5).

## 4 DISCUSSION

Novel findings were that the individual NKA α_1_–α_3_ and β_1_–β_3_ gene transcripts were not changed after 14 days’ oral digoxin intake, whereas the total β gene transcripts in resting muscle were increased after digoxin. This indicates an overall stimulatory effect of digoxin on NKA β gene expression. The acute exercise protocol elevated only α_1_ and β_3_ mRNA, whereas α_3_ mRNA declined and each of α_2_, β_1_ and β_2_ mRNA were unchanged from rest. Furthermore, none of the NKA α or β gene responses to exercise were exacerbated or impaired by digoxin. As anticipated, the protein abundances of individual NKA isoforms in muscle were unaffected by either digoxin or acute exercise, with the exception of a decline in α_2_ protein at 3 h post‐exercise.

### 4.1 Increased total NKA β gene expression with digoxin

We explored whether digoxin ingestion over 14 days may increase NKA gene expression in human muscle, finding no changes in the individual α_1_–α_3_ or β_1_–β_3_ isoform gene transcripts. However, the NKA total β mRNA was increased by 60% in healthy resting muscle after digoxin, indicating overall increased transcription and/or reduced degradation of NKA β gene transcripts. As digoxin binds to and inhibits the α protein subunit, an increased NKA total α mRNA might similarly be anticipated. However, although the total α mRNA appeared higher in DIG than CON both when calculated using the $2^{-\Delta CT}$ data summed from each of α_1_–α_3_ (47%) and also when summed after then normalising these against placebo (94%), these did not differ significantly. The lack of change in individual isoform mRNA with digoxin is unlikely due to inadequate digoxin intake and NKA‐binding in these participants. As we also did not measure intracellular [Na^+^], we cannot say whether the lack of response of individual NKA genes to digoxin was due to inadequate inhibitory effects of digoxin on in vivo NKA activity in muscle. However, our calculations of increased NKA total β isoform gene transcripts is consistent with the hypothesis that inhibition of a small NKA fraction in muscle by digoxin would stimulate increased NKA β isoform mRNA expression in muscle. The resting serum digoxin reached 0.8 nmol L^−1^ (∼0.62 ng mL^−1^) (Sostaric et al., 2022), which is within the optimal therapeutic range (Bavendiek et al., 2017), indicating that the dosing regime was effective in achieving the desired concentration consistent with clinical use. However, ∼1 nmol L^−1^ digoxin is a low dose compared to the experimental dosage of digoxin or ouabain (µM or mM) utilised in non‐human tissues, as described below. Thus, it is possible that an optimal clinical dose for human treatment was still suboptimal for inducing changes in NKA gene expression in muscle. Another possibility is that an initial digoxin‐induced upregulation of individual NKA isoform gene transcripts occurred, but that these were only transient and hence no longer detected after 14 days, with the exception of the calculated total β mRNA, where residual changes were still large enough to be detected when summed. A time‐course study would be required to ascertain whether transient effects might have occurred. The observed statistical power for these mRNA analyses was, however, low, with a small sample size, suggesting that the lack of effect of digoxin might also reflect a Type II error for one or more of these NKA genes.

Findings of cardiotonic steroid effects on mRNA in other species and tissues are varied. In rat outer medullary loop tubules, 100 µM ouabain increased α_1_ and β_1_ mRNA (Rayson, 1993). In rat cultured neonatal ventricular myocytes, 1 mM ouabain increased α_1_–α_3_ mRNA 3–4‐fold and β_1_ 2‐fold (Yamamoto et al., 1993) and these were later shown to occur in a dose‐dependent manner from 10 to 100 µM ouabain (Kometiani et al., 2000). In contrast, exposure of cultured ventricular myocytes from adult rats to 30 µM digoxin for 24 h markedly reduced NKA α_1_–α_3_ mRNA expression (Gan et al., 2009). Also in adult rats, digoxin and ouabain infusion affected NKA isoform gene expression in a tissue‐specific manner and also differentially within the same tissue; for example in the myocardium, both ouabain (20 µg kg^−1^ per day) and digoxin (32 µg kg^−1^ per day) infusion over 6 weeks increased α_3_ mRNA whilst ouabain decreased α_1_ mRNA expression (Wang et al., 2000). Incubation of rat EDL muscle for 2 h in 1 mM ouabain had no effect on α_1_–α_3_ or β_1_ mRNA, but reduced β_2_ and β_3_ mRNA by 76% and 92%, respectively (Murphy, Macdonald, et al., 2006). These studies finding that cardiac glycosides differentially affect NKA isoform mRNA and transcription rates used different species, tissues and cell preparations, with different glycoside concentrations and exposure times and yielded conflicting findings. Hence, it is difficult to compare their findings to ours in skeletal muscle in healthy humans. Inhibition of NKA α_1_ by cardiotonic steroids in other tissues demonstrated the NKA was also involved in signal transduction, protein–protein interactions and intracellular signalling functions via Src‐dependent and ‐independent pathways (Aperia et al., 2016; Cui & Xie, 2017). As we did not measure receptors and other proteins involved in these pathways, we cannot ascertain whether such signalling also occurred in human skeletal muscle. Whilst NKA α_4_ mRNA was earlier detected in human skeletal muscle (Keryanov & Gardner, 2002; Shamraj & Lingrel, 1994), other studies in human muscle reported either extremely low expression levels and being undetectable in a majority of samples (Nordsborg et al., 2005) or just being undetectable (Murphy, Petersen, et al., 2006). Whether digoxin itself might affect α_4_ mRNA expression was not determined here and could be explored in future investigations.

One important additional argument that an inadequate digoxin intake and NKA‐binding is not a likely cause for the lack of change was that the muscle total NKA content (total α protein content) was maintained, not reduced, after 14 d in these participants (Sostaric et al., 2022). We previously reported that digoxin surprisingly did not reduce either the resting muscle [^3^H]ouabain binding site content, a measure of NKA content, or the maximal in vitro K^+^‐stimulated 3‐*O*‐MFPase activity, a marker of NKA activity in these participants, with both these measures instead unchanged (Sostaric et al., 2022). However, incubation of muscle in Digibind to remove all previously bound digoxin demonstrated an additional ∼8% higher [^3^H]ouabain binding after digoxin (Sostaric et al., 2022). This suggested the digoxin challenge actually induced an upregulation of NKA content in muscle. In this context of unchanged functional NKA content and activity in muscle, it is thus not surprising that the individual NKA gene transcripts were not increased after digoxin. This seems the most likely factor responsible for the lack of effect of digoxin here on the individual NKA genes. Nonetheless, the elevated total β transcripts suggested a small, combined stimulatory effect persisted despite this.

The lack of change in NKA isoform protein abundances with digoxin was not surprising. We anticipated based on studies in human myocardium (Schmidt et al., 1991; Schmidt, Allen, et al., 1993) that the [^3^H]ouabain binding site content in skeletal muscle would decline with digoxin, due to a fractional inhibition of NKA by digoxin binding, and further that after removal of bound digoxin by Digibind, the [^3^H]ouabain binding site content would be the same as control values (Sostaric et al., 2022). As such we then also anticipated no change in overall isoform protein abundances would occur with digoxin. However, we found there was an overall 8% increase in [^3^H]ouabain binding site content after the removal of bound digoxin by Digibind (Sostaric et al., 2022). It is reasonable then to expect that an increased overall abundance of α and β subunits should have also occurred. The lack of increase in the α_1_, α_2_, β_1_ or β_2_ isoform protein abundances with digoxin is thus inconsistent with the greater overall [^3^H]ouabain binding site content after digoxin and use of Digibind. The reason for this is unclear but is likely confounded by the typical variability inherent in western blotting for NKA isoform proteins (Christiansen et al., 2018), together with the low sample size and observed power for these measurements. There appear to be no other reported measurements of digoxin effects on NKA isoform protein abundances in skeletal muscle in humans or other species. Thus, further experiments are required to ascertain which of the α isoforms are upregulated at the protein level to account for the increased [^3^H]ouabain binding site content after digoxin. The lack of change in NKA isoform protein abundances with digoxin does not preclude other possible post‐translational changes such as NKA subunit phosphorylation, glutathionylation or ubiquitination, which may potentially affect NKA activity, trafficking or degradation (Helenius et al., 2010; Juel et al., 2015; Pirkmajer & Chibalin, 2016). However, the possible effects of digoxin on these processes in the regulation of NKA in skeletal muscle remain to be determined.

### 4.2 Exercise effects on NKA gene and protein expression

Mild‐to‐moderate cycling for 20 min (33%, 67% ${\dot{V}}_{O_{2}\mathrm{peak}}$) that then continued at high intensity (90% ${\dot{V}}_{O_{2}\mathrm{peak}}$) to fatigue increased both the α_1_ and β_3_ mRNA transcripts at 3 h post‐exercise, and reduced α_3_ mRNA at 67% ${\dot{V}}_{O_{2}\mathrm{peak}}$ and at fatigue. The mechanisms responsible potentially include acute increases in reactive oxygen species, intracellular [Na^+^], cytosolic [Ca^2+^] and reduced membrane potential, which are likely to be greater with higher exercise intensity (Christiansen, 2019). In contrast, no exercise effects were found on the α_2_, β_1_ or β_2_ mRNA. Numerous studies have demonstrated that acute exercise increased NKA gene transcripts in human skeletal muscle (Aughey et al., 2007; Christiansen et al., 2018; Murphy et al., 2008, 2004; Murphy, Petersen, et al., 2006; Nordsborg et al., 2003, 2005; Petersen et al., 2005; Pillon et al., 2020). As an increase in α_1_ mRNA after exercise is a robust finding across multiple studies, this appears to be an obligatory response to exercise. This may be linked with the essential functional role of the α_1_ protein in regulating trans‐membrane Na^+^/K^+^ exchange and membrane potential in resting muscle and hence also its importance in recovery from exercise (McKenna et al., 2024; Radzyukevich et al., 2004, 2013). The submaximal exercise intensities used here, even though exhaustive, might explain the lack of exercise effects on NKA α_2_, β_1_ and β_2_ mRNA. By comparison, we and others previously found the α_1_–α_3_ and β_1_–β_3_ isoform mRNA were increased after brief, intense exercise (Murphy et al., 2004; Petersen et al., 2005, and the α_1–3_ isoform mRNA were elevated after intense, intermittent exercise (Aughey et al., 2007; Murphy et al., 2004, Murphy, Petersen, et al., 2006; Nordsborg et al., 2005; Petersen et al., 2005. Although we previously found α_1_, α_3_ and β_2_ mRNA were each increased after submaximal exercise at ∼75% ${\dot{V}}_{O_{2}\mathrm{peak}}$ (Murphy, Petersen, et al., 2006), the duration of exercise was approximately double that employed here and the intensity during the initial 20 min cycling was less than this level. Thus exercise at intensities exceeding ${\dot{V}}_{O_{2}\mathrm{peak}}$ or submaximal but for more extended durations may be required to stimulate increased α_2_, β_1_ and β_2_ mRNA in muscle. It is unlikely that the time course of NKA gene activation was missed, as most studies report NKA mRNA changes within 0–3 h post‐exercise (Murphy et al., 2004; Murphy, Petersen, et al., 2006; Nordsborg et al., 2003, 2005; Pillon et al., 2020). It is possible that our inability to detect increases with exercise in the NKA α_2_, β_1_ or β_2_ gene transcripts could also be due to individual response and measurement variabilities. A further possible explanation for the increase after exercise in muscle α_1_ mRNA, but not α_2_, β_1_ and β_2_ mRNA, is the relative abundance of the different subunit isoforms at mRNA and protein levels. In human muscle, the α_1_ mRNA expression was 20‐ and 68‐fold lower than of α_2_ and β_1_ mRNA, respectively (Nordsborg et al., 2005). In rat muscle membranes, the molar abundance of α subunit protein was 60–75% less than that of β subunit proteins (Lavoie et al., 1997). Thus, a greater adaptability of α_1_ mRNA with acute exercise could be consistent with low initial mRNA and stimulus to increase α_1_ protein abundance, to regulate basal Na^+^/K^+^ exchange. However, no evidence of increased α_1_ protein after exercise was found here. We recently reported that the muscle [^3^H]ouabain binding site content was increased after only 20–30 min exercise in these individuals and proposed that this might reflect an increased formation of functional NKA complexes from already existing, but non‐bound α and β subunits in muscle that were either inserted into or being trafficked to sarcolemmal/t‐tubular membranes (Sostaric et al., 2022), as reported in other cell types (DeTomaso et al., 1994). It is not yet possible to determine whether this may have indeed occurred. Many studies have investigated differences in NKA isoform localisation in sarcolemmal, t‐tubular and intracellular membranes in skeletal muscle, using membrane purification, fractionation, immunoblotting and also immuno‐histological techniques and furthermore whether exercise might induce their translocation from intracellular to plasma membranes, as recently reviewed (McKenna et al., 2024; Pirkmajer & Chibalin, 2016). Findings suggest that NKA isoform locations and their possible translocation with exercise arguably differ according to species, muscle, fibre type, as well as the techniques used. In rat muscles there is evidence of different localisations for NKA isoforms and also evidence supporting contraction‐induced translocation, but inconsistencies abound, whilst little evidence yet exists in human muscles (McKenna et al., 2024). Our finding of unchanged NKA α_1_, α_2_, β_1_ and β_2_ protein abundances during acute exercise are also consistent with this possibility of new NKA formed from pre‐existing non‐bound isoforms. These also confirm our earlier reports of unchanged NKA α_1_, α_2_, β_1_ and β_2_ protein after brief, exhaustive exercise (Murphy et al., 2004), as well as prolonged submaximal exhaustive exercise (Murphy, Petersen, et al., 2006). Since the western blots were determined using whole muscle homogenates without purification steps, it is unlikely that any NKA proteins that may have been located in membranous structures were lost and not subsequently detected. An exception to the lack of change in NKA isoforms with exercise was a surprising small decline in α_2_ proteins at 3 h post‐exercise, for which the reason is unclear.

Physical activity was restricted for 24 h before each experimental trial, raising the possibility that this short period might have influenced the muscle NKA content, isoform mRNA expression or protein abundances, but this seems unlikely. Although chronic injury or disease that is associated with inactivity reduced muscle NKA content by 20–45% in humans (McKenna et al., 2024), reduced activity induced by lower limb unloading for 23 days did not reduce NKA content or isoform protein abundances in muscle homogenates (Perry et al., 2016). Muscle NKA α_1_, α_2_ and β_2_ protein abundances were also unchanged in type I and II fibres after limb unloading, whilst declines in α_3_ and in β_1_ protein abundances were found in type I and II fibres, respectively (Perry et al., 2016). This suggests that major changes in muscle mRNA are unlikely to be manifest within 24 h of restricted activity. Furthermore, whilst endurance‐trained men exhibited lower muscle NKA α_1_, α_3_, β_2_ and β_3_ mRNA than recreationally trained men (Murphy et al., 2007), high‐intensity training for 3–5 weeks did not change α_1_, α_2_ or β_1_ mRNA expression in resting muscle (Aughey et al., 2007; Nordsborg et al., 2003), although elevations in α_3_ and β_3_ mRNA were found (Aughey et al., 2007). Participants were also asked to maintain their habitual physical activity levels throughout the trial, and although these were not measured, it suggests possible training or detraining effects were unlikely.

In conclusion, 14 days’ oral digoxin did not augment expression of the individual NKA isoform genes in these healthy individuals, although there was evidence of increased total β subunit mRNA expression in resting muscle. Acute exercise increased NKA α_1_ and β_3_ mRNA and depressed α_3_ mRNA in muscle, but did not modify α_2_, β_1_ and β_2_ mRNA. It is likely that exercise of higher intensity, or of much longer duration is required to stimulate increased expression of these gene transcripts in muscle. None of the NKA gene transcript changes with exercise were exacerbated or impaired by digoxin. As anticipated, digoxin did not decrease, or acute exercise increase NKA isoform protein abundances. These findings suggest that provided functional content of NKA is preserved in muscle, digoxin inhibition of NKA, whilst increasing total β transcripts, does not markedly increase NKA gene expression in muscle or evoke greater responses in NKA genes with exercise.

## AUTHOR CONTRIBUTIONS

Michael J. McKenna, Henry Krum and Rodney J. Snow conceived and designed the research. Aaron C. Petersen, Xiaofei Gong, Simon Sostaric, Craig A. Goodman, Andrew Garnham, Juan Aw, Collene H. Steward, Kate T. Murphy, Kate A. Carey, David Cameron‐Smith, Rodney J. Snow and Michael J. McKenna conducted the experiments. Xiaofei Gong, Aaron C. Petersen and Michael J. McKenna analysed data. Michael J. McKenna, Aaron C. Petersen and Xiaofei Gong performed the statistical analyses. Michael J. McKenna, Aaron C. Petersen and Xiaofei Gong wrote the initial manuscript draft. All authors read and approved the final manuscript, except Henry Krum (deceased) and Xiaofei Gong (departed university and now uncontactable) who each read earlier versions. All authors agree to be accountable for all aspects of the work in ensuring that questions related to the accuracy or integrity of any part of the work are appropriately investigated and resolved, with the exceptions of Henry Krum and Xiaofei Gong, as per above.

## CONFLICT OF INTEREST

The authors report no conflicts of interest.

## Supporting information

Table S1. Observed power for Na^+^,K^+^‐ATPase α and β isoform mRNA expression and protein abundances for treatment (digoxin, control) and time (rest, 67% ${\dot{V}}_{O_{2}\mathrm{peak}}$, fatigue and 3 h post‐exercise). Observed power was calculated using IBM SPSS Statistics Version 27.

## Data Availability

All data cited in this paper are available on Open Science Forum and can be accessed at: https://osf.io/ku82x/?view_only=370e1f3f0da248bba9b6d28fc6a895d6.
