# Supplementary material for: Digoxin and exercise effects on skeletal muscle Na+,K+‐ATPase isoform gene expression in healthy humans
Source: Exp Physiol. 2024 Sep 2;109(11):1909–21. doi: 10.1113/EP091962 (PMC11522823; doi:10.1113/EP091962)
Supplement: Supplementary file 1 — Table S1. Observed power for Na+,K+‐ATPase α and β isoform mRNA expression and protein abundances for treatment (digoxin, control) and time (rest, 67% V˙O2peak, fatigue and 3 h post‐exercise). Observed power was calculated using IBM SPSS Statistics Version 27. [file EPH-109-1909-s001.pdf]

Supplementary Table 1.

Observed power for Na^+,K^+-ATPase α and β isoform mRNA expression and protein abundances for treatment (digoxin, control) and time (rest, 67%VO_{2peak}, fatigue and 3 h post-exercise). Observed power was calculated using SPSS Ver. 27.

**Variable**	**Treatment**	**Time**
α_1 mRNA	0.158	0.252
α_2 mRNA	0.109	0.148
α_3 mRNA	0.202	0.337
β_1 mRNA	0.152	0.108
β_2 mRNA	0.051	0.193
β_3 mRNA	0.354	0.635
α_1 Protein	0.062	0.069
α_2 Protein	0.051	0.342
β_1 Protein	0.084	0.177
β_2 Protein	0.050	0.283
